# Supplementary material for: The interactome of intact mitochondria by cross-linking mass spectrometry provides evidence for coexisting respiratory supercomplexes
Source: Mol Cell Proteomics. 2017 Dec 8;17(2):216–32. doi: 10.1074/mcp.RA117.000470 (PMC5795388; doi:10.1074/mcp.RA117.000470)
Supplement: Supplemental Data [file supp_RA117.000470_133922_0_supp_23978_fzffwf.zip › spectra_annotation/mito_spectra_annotation/310-2-6-1-8-1.pdf]

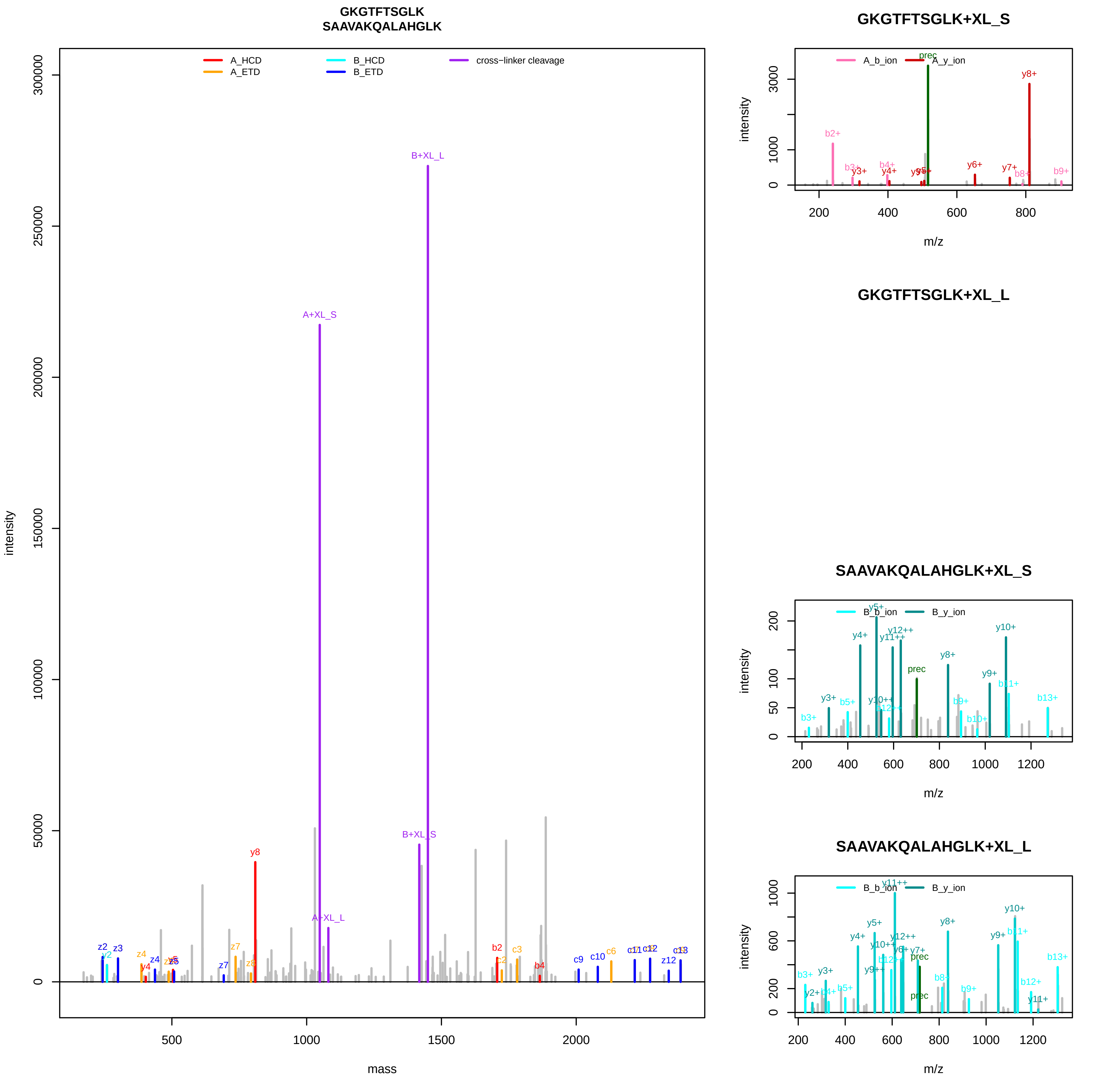

GKGTFTSGLK
SAAVAKQALAHGLK
A_HCD
A_ETD
B_HCD
B_ETD
cross−linker cleavage
B+XL_L
A+XL_S
B+XL_S
A+XL_L
y8
intensity
mass
GKGTFTSGLK+XL_S
A_b_ion
A_y_ion
prec
y8+
b2+
y6+
y7+
b9+
m/z
GKGTFTSGLK+XL_L
SAAVAKQALAHGLK+XL_S
B_b_ion
B_y_ion
y5+
y4+
y12++
y11++
y10+
y8+
prec
y9+
b11+
b13+
y3+
b5+
b3+
b9+
b10+
SAAVAKQALAHGLK+XL_L
y11++
y10+
y8+
y9+
b11+
b13+
b12+
y11+
